# Supplementary material for: Modelling Functional Thyroid Follicular Structures Using P19 Embryonal Carcinoma Cells
Source: Cells. 2024 Nov 7;13(22):1844. doi: 10.3390/cells13221844 (PMC11593046; doi:10.3390/cells13221844)
Supplement: Supplementary file 1 [file cells-13-01844-s001.zip › cells-3214702-supplementary.pdf]

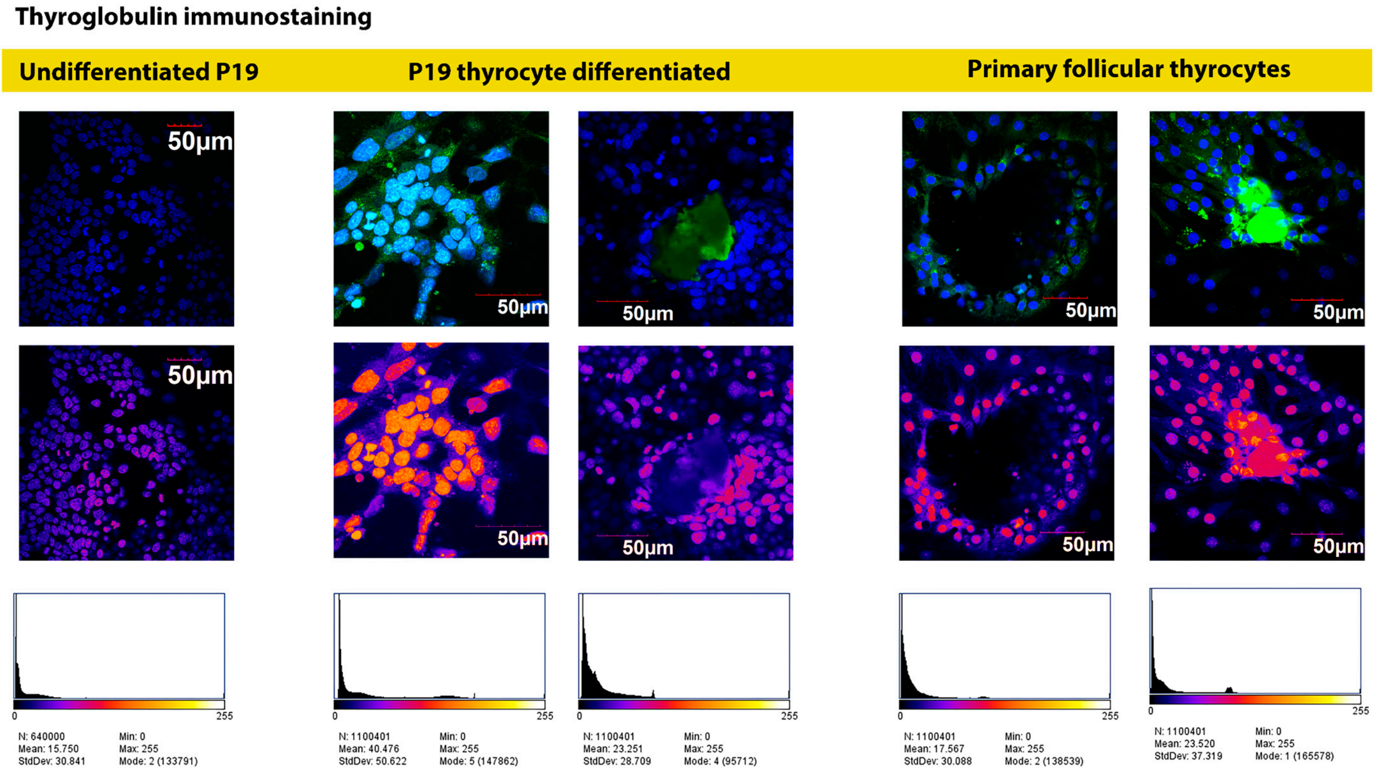

Figure S1. Heat maps and histograms of thyroglobulin immunostaining for Figure 4.A. The top panel represents images originally from Fig4.A., the middle panel represents the heatmaps, and the bottom panel represents the histograms. Heatmap is a colored map shown in a 2-dimensional manner to visualize the data density. The high density of data shifts towered white color (saturation pixels), and the low density of data shifts towered blue color. Histogram is a graphical representation of the distribution of pixel values across the available bit-depth of an image. The image on the left was originally from Fig 4.A. panel (top left). The two images in the middle were originally from Fig 4.A. panel (top and lower middle). The two images on the right were originally from Fig 4.A. panel (top and lower right).

Thyroglobulin immunostaining

P19-siRNA control thyrocyte differentiated (Day6)

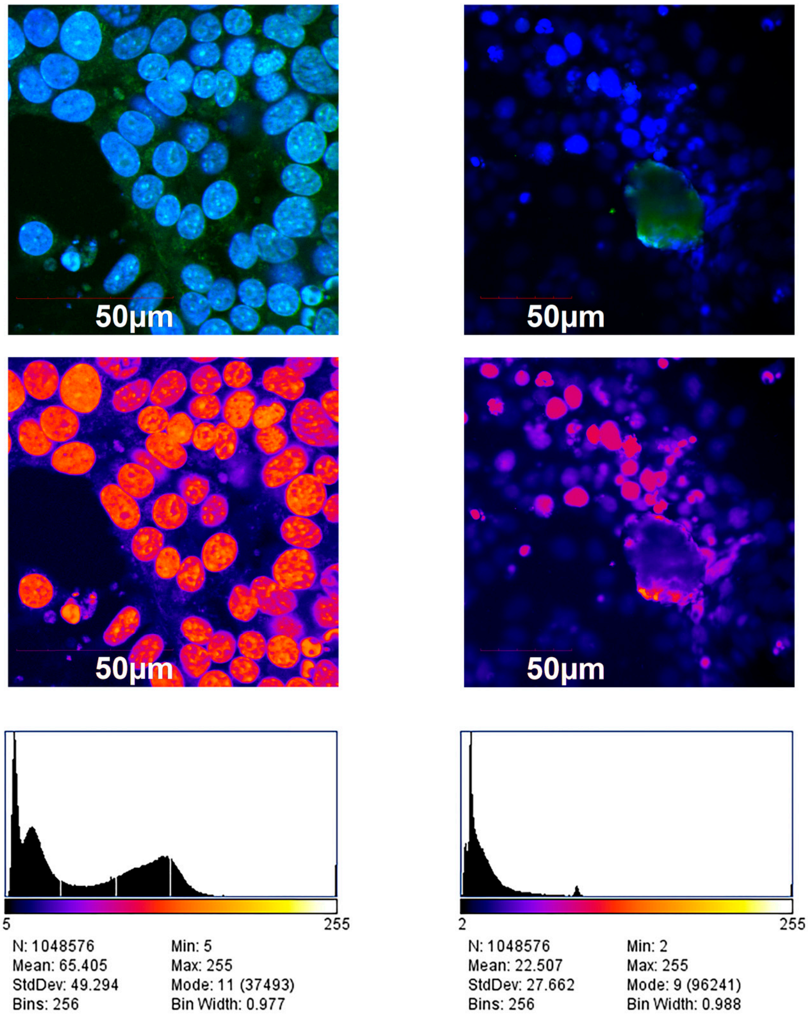

Figure S2. Heat maps and histograms of TG. The right image was originally in Fig 7.B. panel on the left. The top panel represents images originally from Fig .7.B., the middle panel represents the heatmaps, and the bottom panel represents the histograms. The left image was originally in Fig 7.B. panel in the middle.

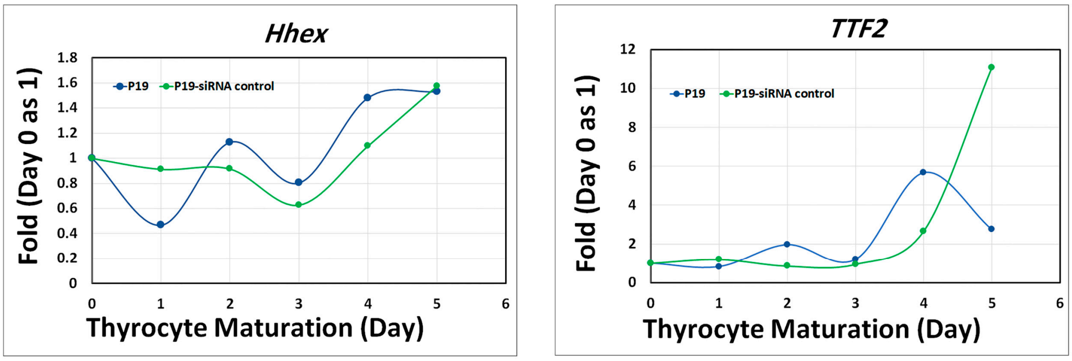

Figure S3 Expression of key transcription factors *TTF2, Hhex* patterns of wild type P19 and P19-siRNA control during maturation
